# Odorant-binding protein 84a-1 mediates detection of Guire No.82 mango volatiles in *Bactrocera dorsalis*: from structural analysis to behavioral validation

**DOI:** 10.3389/finsc.2025.1712208

**Published:** 2026-01-06

**Authors:** Peng Peng, Yingying Tang, Shian Yang, Yingxi Mo, Guodi Huang, Yongsen Chen

**Affiliations:** 1 Guangxi Subtropical Crops Research Institute, Guangxi Academy of Agricultural Sciences, Nanning, China; 2 Guangxi Zhuang Autonomous Region Engineering Research Center of Green and Efficient Development for Mango Industry, Nanning, China

**Keywords:** *Bactrocera dorsalis*, behavioral validation, mango volatiles, molecular dynamics, odorant-binding protein

## Abstract

*Bactrocera dorsalis* is a serious pest of mango, causing heavy losses during fruit ripening. Odorant-binding proteins (OBPs) are pivotal in insect olfaction, facilitating the detection of host plant volatiles. In this study, we focused on *OBPs* that mediate responses to volatiles from the Guire No.82 mango. Quantitative real-time PCR showed cultivar-dependent expression of six antennal OBP genes. Among these, *BdorOBP84a-1* was highly expressed in adults fed on Guire No.82. The 3D structure of BdorOBP84a-1 was predicted with AlphaFold3, showing six α-helices and three disulfide bonds forming a hydrophobic pocket. Molecular docking and 100-ns MD simulations indicated strong and stable binding of sesquiterpenes. Caryophyllene and ledene showed the lowest binding free energies (-31.87 and -30.62 kcal/mol) and stable RMSD/RMSF values. Key residues, including PHE95, ILE109 and PHE133, contributed to binding through hydrophobic and aromatic interactions. Y-tube behavioral assays validated these computational predictions. Caryophyllene attracted males at very low (0.01%) and high (10%) doses, females responded selectively at 0.1% and 1%, ledene attracted males at 0.1% and 10%, These findings suggest that BdorOBP84a-1 mediates recognition of mango sesquiterpenes and that caryophyllene and ledene are promising leads for new attractants in *B. dorsalis* management.

## 1 Introduction

Insects rely primarily on their olfactory system to locate food and host plants. They detect plant-derived volatile organic compounds (VOCs) through a specialized sensory system (1, 2). Within this system, odorant-binding proteins (OBPs) serve as soluble carriers. They transport hydrophobic odorant molecules across the sensillar lymph and deliver them to odorant receptors (ORs) located on olfactory sensory neurons (OSNs) (3, 4). This process triggers neuronal activation, chemical signals are converted into electrical impulses, these impulses are transmitted to the insect brain, thereby enabling odor recognition, thus playing a crucial role in peripheral olfaction (5).

OBPs were first identified in the antennae of the moth *Antheraea polyphemus* (Lepidoptera: Saturniidae) (6), they are small (~140 amino acids), water-soluble proteins (7). Classical OBPs contain six conserved cysteine residues that form three disulfide bonds, these bonds stabilize the α-helical structure and create a hydrophobic binding pocket for odorants (8, 9). Most OBPs show high expression in antennae and other chemosensory tissues, acting as primary molecular recognition elements in the olfactory pathway (1). Functional studies show that each OBP has distinct ligand-binding spectra, for example, DcitOBP3 in *Diaphorina citri* binds multiple odorants (10), whereas CpomPBP2 in the *Cydia pomonella* has high affinity for 1-dodecanol (11).

Traditional OBP-ligand interaction studies rely on fluorescence competition assays (12), These methods require protein purification and labeling, which are both time-consuming and expensive. Advances in bioinformatics and structural biology have led to “reverse chemical ecology” (13). This approach uses the binding profiles of OBPs to identify attractants and repellents more efficiently. Molecular docking has become a widely used computational method to predict protein-ligand interactions (14, 15). Its accuracy depends on the availability of reliable protein structures, the release of AlphaFold3 in 2024 has enabled near-experimental accuracy in 3D structure prediction (16), providing a solid foundation for virtual screening. Molecular docking has been applied across many insect orders. Molecular docking is commonly used to model OBP–ligand interactions and identify behaviorally active volatiles (17, 18), examples include *Harmonia axyridis* in Coleoptera, *Rhodnius prolixus* in Hemiptera, *Megachile saussurei* in Hymenoptera, and *Locusta migratoria* in Orthoptera, where OBPs were docked with host, sex pheromone, floral, or general odorant molecules, respectively (19–22).

Docking provides important insights but only captures a static view of ligand-protein binding. Molecular dynamics (MD) simulations complement docking by testing the stability, flexibility, and binding energy of complexes over time (23, 24). A standard workflow often combines structural modeling or AlphaFold prediction, docking-based ligand screening, 100–200 ns explicit-solvent MD simulations, free energy calculations, and hotspot residue analysis. These predictions are then validated by mutagenesis or physiology. For example, in *Athetis lepigone*, AlepPBP2 and AlepPBP3 showed key residues involved in phoxim binding (25); *Plutella xylostella* PxylOBP1 stably bound ethyl and methyl gallate (26); this workflow has also clarified OBP-odorant interactions in other insects. For example, in *Drosophila melanogaster*, Thr57 in the OBP76a binding pocket forms a critical hydrogen bond with alcohols (27); and in *Agrilus mali*, hydrogen bonding dominates the interaction between AmalOBP8 and geranyl formate, with Trp106 in the binding pocket playing a pivotal role (28).

Mango (*Mangifera indica* L.), belonging to the family Anacardiaceae and the genus Mangifera, is one of the most important tropical fruits. It is widely grown in India, China, Thailand, Myanmar, Mexico, and Brazil (29). Guangxi is one of the leading mango producing regions in China (30), both in cultivation area and yield. Several cultivars are common, including Tainong No.1, Jin Huang, Ketti, Gui Fei, Guire No.82, and Guire No.10. Among them, Guire No.82 covers more than 15,000 hectares in Baise City. It is well adapted to the subtropical climate and resistant to abiotic stress. At ripeness, it produces a strong aroma and contains more than 20% soluble solids, which makes it highly attractive to insect pests (31).

*Bactrocera dorsalis* (Hendel) (Diptera: Tephritidae), is the most destructive pest of mango fruit at the ripening stage (32). Its larvae feed directly on the pulp, causing severe economic loss. Current control strategies mainly rely on attract-and-kill techniques targeting adult flies, with methyl eugenol (ME) widely used as a male-specific lure. However, ME-based approaches suffer from several drawbacks, including narrow target specificity, limited field persistence, and potential carcinogenicity to mammals (33). In *B. dorsalis*, genome-wide annotation identified 49 putative OBPs, of which six are highly expressed in antennae (34).Silencing OBP genes can significantly attenuate insect behavioral responses to semiochemicals. In *Bactrocera dorsalis*, silencing BdorOBP2 significantly reduced male responses to methyl eugenol (35). These findings highlight the critical role of OBPs in odor-mediated behaviors and suggest that they may serve as promising molecular targets for the development of effective lure-based pest control strategies (36).

To date, no study has looked at how *B. dorsalis* OBPs interact with VOCs from the Guire No.82 mango. Our study aims to do this by: (i) finding candidate OBPs expressed after feeding on Guire No.82, (ii) building 3D models with AlphaFold3, (iii) using docking, MD, and MM/PBSA to characterize OBP–VOC binding and evaluate stability and energetics, and (iv) conducting behavioral assays of *B. dorsalis* to test responses to VOCs. These results will help us understand how *B. dorsalis* finds hosts and may guide the design of safe, species-specific attractants for pest control.

## 2 Materials and methods

### 2.1 Insect rearing

Adult *Bactrocera dorsalis* were obtained from Guangxi Subtropical Crops Research Institute (22.90° N, 108.33° E), Nanning, Guangxi, China, and continuously reared under controlled environmental conditions in an artificial climate chamber. The rearing conditions were maintained at 25 ± 1 °C, relative humidity of 70 ± 10%, and a photoperiod of 16:8 h (light: dark). Before starvation, adult flies were maintained on an artificial diet containing honey, sugar, yeast powder, and vitamin C, whereas larvae were reared on a diet composed of corn, wheat germ flour, yeast powder, agar, sugar, sorbic acid, vitamin C, linoleic acid, and filter paper.

### 2.2 Experimental treatments

Healthy 3-day-old adult *B. dorsalis* (n=3 biological replicates, 10 insects each) were starved for 24 h prior to the experiment and then transferred into cylindrical plastic rearing containers (radius 4 cm, height 15 cm). Each container was supplied with 4 cm × 4 cm × 4 cm cubes of ripe mango pulp from six cultivars: Tainong No.1, Gui Fei, Guire No.82, Guire No.10, Keitt and Jin Huang. Each treatment was conducted in triplicate. After 24 h of feeding on the mango pulp, the adults were dissected to collect antennae for subsequent analyses.

### 2.3 Quantitative real-time PCR assay

Total RNA was extracted from the adult antennae (10 pairs) of each treatment group using TRIzol Reagent (Invitrogen, USA), followed by DNase I treatment to remove genomic DNA contamination. RNA quality was assessed by agarose gel electrophoresis and NanoDrop spectrophotometry. First-strand cDNA was synthesized using the HiScript II Reverse Transcriptase Kit (Vazyme, China).

Six *B. dorsalis* OBP genes with high antennal expression were selected as targets, with α-tubulin as the reference gene. Sequence data were retrieved from the NCBI database, and specific primers were designed using Primer Express 3.0 and synthesized commercially. The corresponding accession numbers and primer information are listed in **Supplementary Table S1**. RT-qPCR was performed using SYBR Green chemistry in a total reaction volume of 20 μL, containing 1 μL of cDNA, 0.4 μL each of F/R primers (10 μM), 10 μL of Universal SYBR qPCR Master Mix (Vazyme, China), and 8.2 μL of ddH_2_O, reaction setting were: 95 °C for 3 min, followed by 40 cycles of 95 °C for 15 s, 58 °C for 15 s, and 72 °C for 20 s. Three biological replicates and three technical replicates were included for each treatment. Relative expression levels were calculated using the 2^^−ΔΔCt^ method, using α-tubulin as reference genes.

### 2.4 Structural modeling with AlphaFold3

The *BdorOBP* genes that exhibited high expression levels while the insects were feeding on Guire No. 82 mango were selected for structural modelling. The 3D structures of the corresponding proteins were predicted using AlphaFold3 (https://alphafoldserver.com/) through a Google account. The predicted models were subsequently visualized, and their secondary structures were analyzed using PyMOL software (v3.1). To assess model reliability, Ramachandran plot analysis was performed with the PyMod 3 plugin to evaluate the stereochemical quality and structural accuracy of the predicted proteins.

### 2.5 Molecular docking

The CAS numbers of the volatile ligands from Guire Mang No. 82 are listed in **Supplementary Table S2**, and their corresponding 3D structures were obtained from PubChem (https://pubchem.ncbi.nlm.nih.gov/). Receptor structures were prepared by removing crystallographic waters/ions and adding polar hydrogens, energy-minimized, and converted to PDBQT. Docking was performed with AutoDock Vina 1.2.3. Search boxes were centered on the predicted pocket (110 × 110 × 110 Å, spacing 0.375 Å) was centered to encompass the predicted active binding site. Binding affinities were evaluated based on docking scores. The top-ranked poses were inspected and visualized in PyMOL tools (v3.1).

### 2.6 Molecular dynamics simulations

MD simulations were conducted with GROMACS package (v2022) using CHARMM36 for proteins and CGenFF server parameters for ligands. Complexes were solvated in TIP3P water with 1.0 nm padding and neutralized with counter ions. Energy minimization and equilibration were performed under periodic boundary conditions with the Particle Mesh Ewald method for long-range electrostatics (1.0 nm cutoff) and SHAKE constraints on bonds involving hydrogen. Production runs were carried out for 100-ns in an NPT ensemble at 310 K and 1 atm pressure using a 1 fs integration step.

Trajectory analyses included root-mean-square deviation (RMSD), root-mean-square fluctuation (RMSF), radius of gyration (Rg), solvent-accessible surface area (SASA), and Gibbs free energy. Binding free energies were estimated by MM-PBSA using the g_mmpbsa package.

### 2.7 Y-tube olfactometry behavioral assay

Behavioral assays were conducted to evaluate the responses of *B. dorsalis* adults to caryophyllene (Macklin, China, Purity: 99%), ledene (Macklin, China, Purity: 95%), which were selected for testing based on their superior performance in molecular dynamics simulations. ME (Macklin, China, Purity: 99%) was used as a positive control. All assays were performed evaluated using a glass Y-tube olfactometer (common stem 20 cm, arms 15 cm, 30° angle, 1 cm inner diameter). Filtered, humidified air was supplied at 300 mL·min^−1^ per arm. Test solutions (10%, 1%, 0.1% and 0.01%) (v/v) were prepared in paraffin oil and 10 μl applied to filter paper strips; paraffin oil as control. For each dose, three independent replicates were performed, with each replicate comprising 20 adult flies (10 females and 10 males). A choice was recorded when a fly remained ≥ 30 s in an arm within 5 min; non-responders were recorded and reported. Treatment and control sides were alternated between replicates.

### 2.8 Statistical analysis

Statistical analyses were performed using SPSS 16.0 (SPSS Inc., Chicago, IL, USA). qPCR data were analyzed using one-way ANOVA followed by Tukey’s HSD *post hoc* test. In the Y-tube olfactometer assays, insect choice data were evaluated using binomial tests. All quantitative results are presented as means ± SEM from at least three independent experiments.

## 3 Results

### 3.1 Real-time quantitative PCR results analysis

**Figure 1:**
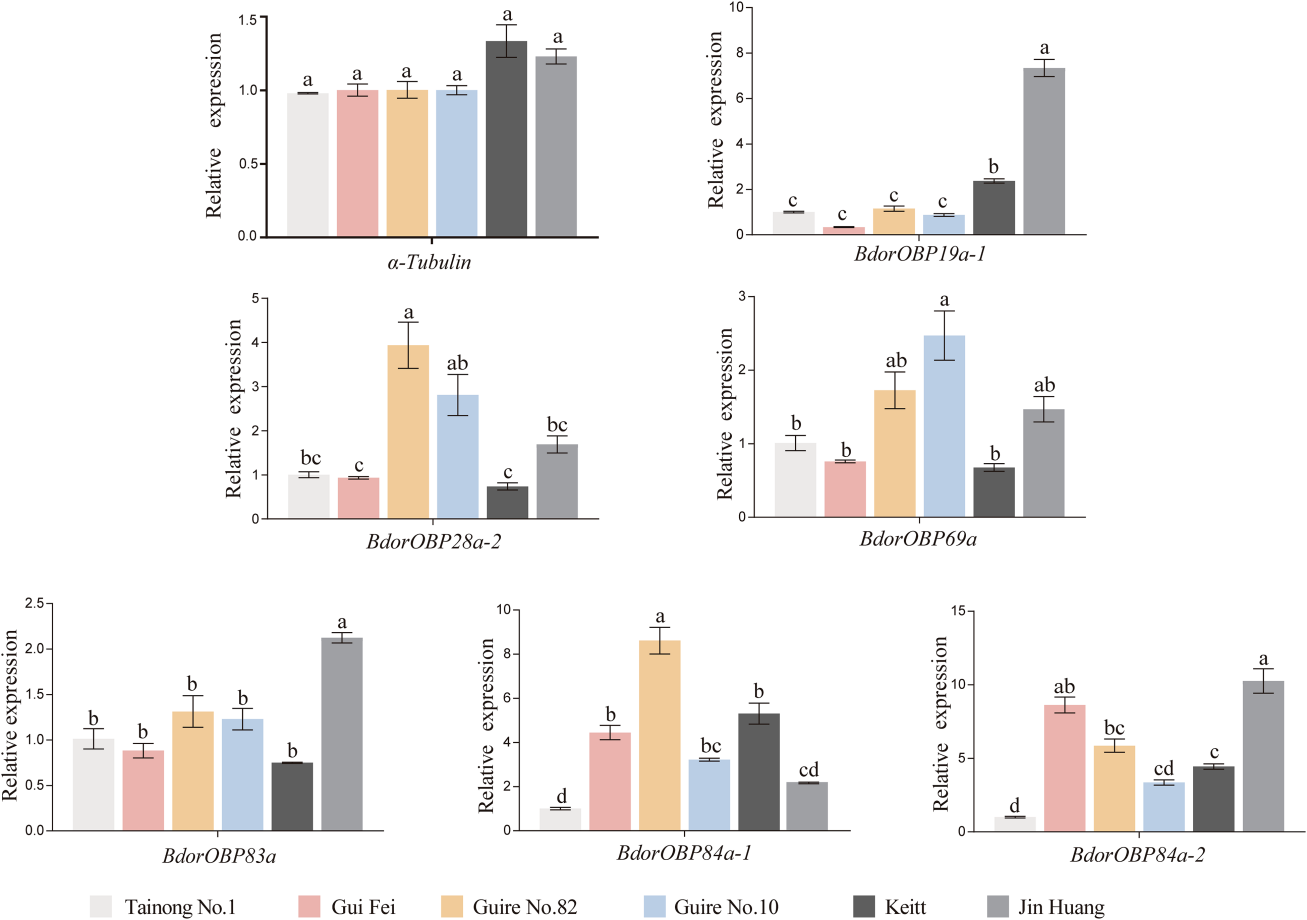
Relative expression levels of *BdorOBP* in *B*. *dorsalis*. Error bars indicate the standard error of the mean. Statistical analysis was performed using one-way ANOVA followed by Tukey’s HSD *post hoc* test, and different lowercase letters above each bar denote significant differences (*p* < 0.05).

Quantitative real-time PCR analysis was performed using cDNA from *B. dorsalis* adults fed on different mango cultivars, targeting six genes with high antennal expression (**Figure 1**). Relative expression was normalized against the *α-tubulin* gene, and expression levels were compared relative to the Tainong No.1 control group. The results showed that the expression of the reference gene *α-tubulin* remained stable across all treatments. Among the target genes, *BdorOBP19a-1* and *BdorOBP83a* were most highly expressed in the Jin Huang group compared with the Tainong No.1 group (*p* < 0.05). Meanwhile, *BdorOBP28a-2* exhibited significantly higher expression in adults fed on Guire No.82 and Guire No.10 (*p* < 0.05). In addition, *BdorOBP69a* showed the highest expression in the Guire No.10 group, and *BdorOBP84a-2* was most highly expressed in the Jin Huang and Gui Fei groups. In contrast, *BdorOBP84a-1* exhibited the highest relative expression in the Guire No.82 group, with significant differences compared to the other five treatments (*p* < 0.05).

### 3.2 Modeling and model evaluation

**Figure 2:**
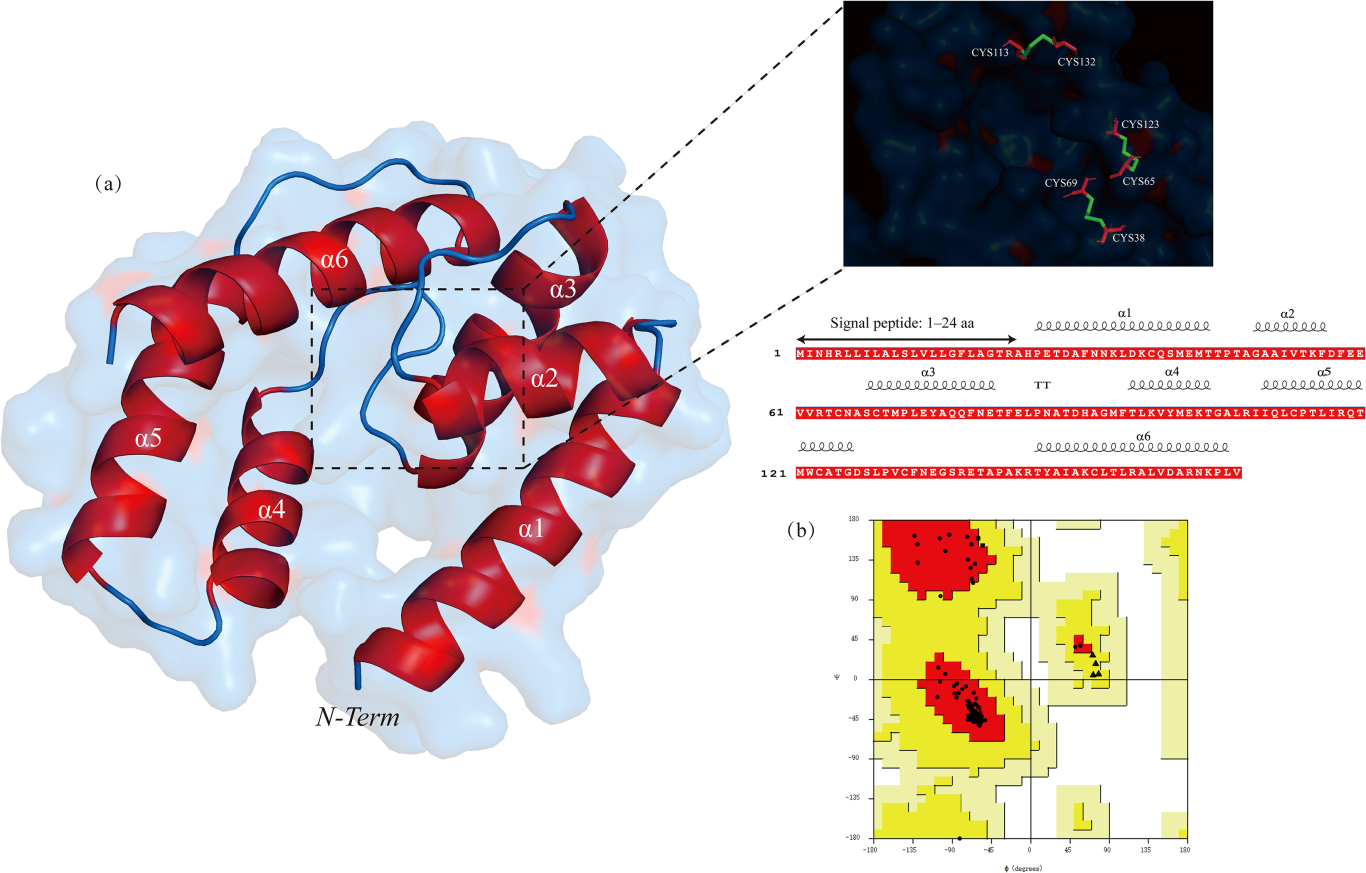
**(a)** Predicted 3D structure and sequences of the BdorOBP84a-1with α-helices shown in red, disulfide bond diagram of BdorOBP84a-1, in which cysteine residues are represented as red sticks and disulfide bonds as green lines. **(b)** Ramachandran plot of the BdorOBP84a-1 protein model showing the most favored (red), additionally allowed (yellow), generously allowed (light yellow), and disallowed (white) regions. Triangles designate glycine residues, squares represent proline residues, and circles indicate other amino acid residues.

BdorOBP84a-1, which showed high expression in *B. dorsalis* adults fed on Guire No.82, was selected as the target protein. The presence of a signal peptide was analyzed using SignalP 6.0 (https://services.healthtech.dtu.dk/service.php?SignalP-6.0) and verified using the UniProt database, confirming an N-terminal signal peptide spanning residues 1–24. Therefore, the signal peptide region was removed and subsequent structural analyses were performed using the mature protein sequence. The 3D structure of BdorOBP84a-1 was predicted with the AlphaFold3 online server based on the signal-peptide–deleted sequence. The predicted model indicated that *BdorOBP84a-1* consisted of 143 amino acid residues, including six α-helices (85 residues in total) and no β-sheets. Seven cysteine residues were identified, forming three disulfide bonds (CYS113 - CYS132, CYS38 - CYS69, and CYS65 - CYS123) (**Figure 2a**). All residue numbers reported in the structural description correspond to the original full-length amino acid sequence prior to signal peptide removal.

The stereochemical quality of the model was evaluated using a Ramachandran plot. Generally, a model is considered reliable if the sum of residues in the favored and additionally allowed regions exceeds 90% of all residues. The Ramachandran analysis of BdorOBP84a-1 (**Figure 2b**) showed 143 amino acid residues, comprising two terminal residues, four glycines, eight prolines, and 129 remaining residues. Among these, 125 residues (96.9%) were in the most favored regions and 4 residues (3.1%) were in additionally allowed regions, accounting for 100% of evaluated residues, indicating that the predicted model was acceptable for subsequent structural analysis.

### 3.3 MD of BdorOBP84a-1-ligand complex

**Figure 3:**
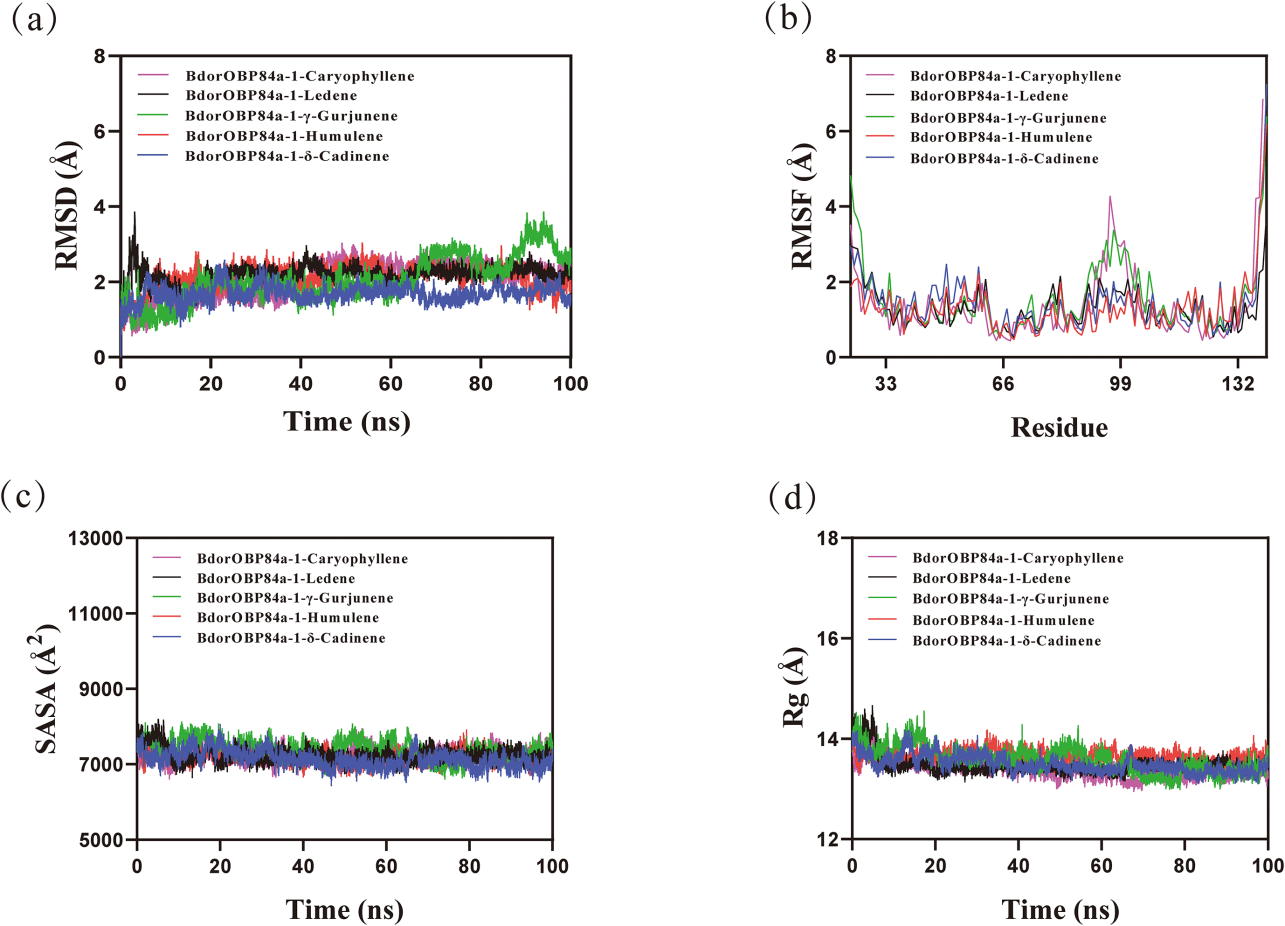
**(a)** Root-mean-square deviation (RMSD) of BdorOBP84a-1-ligand complexes during the 100 ns molecular dynamics (MD) simulations. **(b)** The root-mean-square fluctuation(RMSF) (MD) simulations. **(c)** The solvent-accessible surface area (SASA). **(d)** The radius of gyration (Rg).

Binding affinities between BdorOBP84a-1 and Guire No.82 mango volatile compounds were evaluated using AutoDock Vina. The results (**Supplementary Table S2**) showed that BdorOBP84a-1 exhibited strong affinity for alkenes. Ligands with binding energies below -7.0 kJ/mol were selected as candidates for 100-ns molecular dynamics (MD) simulations with BdorOBP84a-1. As shown in the RMSD plot (**Figure 3a**), except for the BdorOBP84a-1-γ-gurjunene complex, all systems achieved equilibrium after 65 ns. The BdorOBP84a-1-caryophyllene complex stabilized after 65 ns, fluctuating at 2.4 Å. The BdorOBP84a-1-ledene complex stabilized at 2.3 Å after 65 ns, while the BdorOBP84a-1-humulene complex maintained fluctuations below 1.9 Å between 65–85 ns, followed by a slight upward trend thereafter. The BdorOBP84a-1 -δ-cadinene complex reached equilibrium after 70 ns and fluctuated stably around 1.6 Å. These results suggest that ledene, humulene, δ-cadinene, and caryophyllene exhibit relatively high stability when bound to BdorOBP84a-1.

Root mean square fluctuation (RMSF) analysis was performed to evaluate residue flexibility (**Figure 3b**). Across the five ligand-protein complexes, overall fluctuation trends were relatively consistent: the third α-helix exhibited the lowest energy and highest stability, whereas larger fluctuations were observed in α-helices four and five. Notably, the RMSF values of the ledene, humulene, δ-cadinene, and caryophyllene complexes remained mostly below 2 Å, indicating lower flexibility and higher structural stability compared to the γ-gurjunene complex.

The solvent-accessible surface area (SASA) was further analyzed as an indicator of the solvent-exposed surface of the protein. For the same four complexes, the SASA values displayed slight variations throughout the simulations (**Figure 3c**). These results suggest that ligand binding can alter the local microenvironment of the binding pocket, leading to modest changes in solvent exposure of the protein-ligand interface.

The radius of gyration (Rg) is a measure of the overall structural compactness and is widely used to evaluate conformational changes in proteins. For the BdorOBP84a-1 complexes with ledene, humulene, δ-cadinene, and caryophyllene, the Rg profiles exhibited only minor fluctuations during the course of the simulation, indicating that small conformational adjustments occurred within the ligand-protein complexes over time (**Figure 3d**).

### 3.4 Binding modes and conformational stability analysis of BdorOBP84a-1-ligand complexes

**Figure 4:**
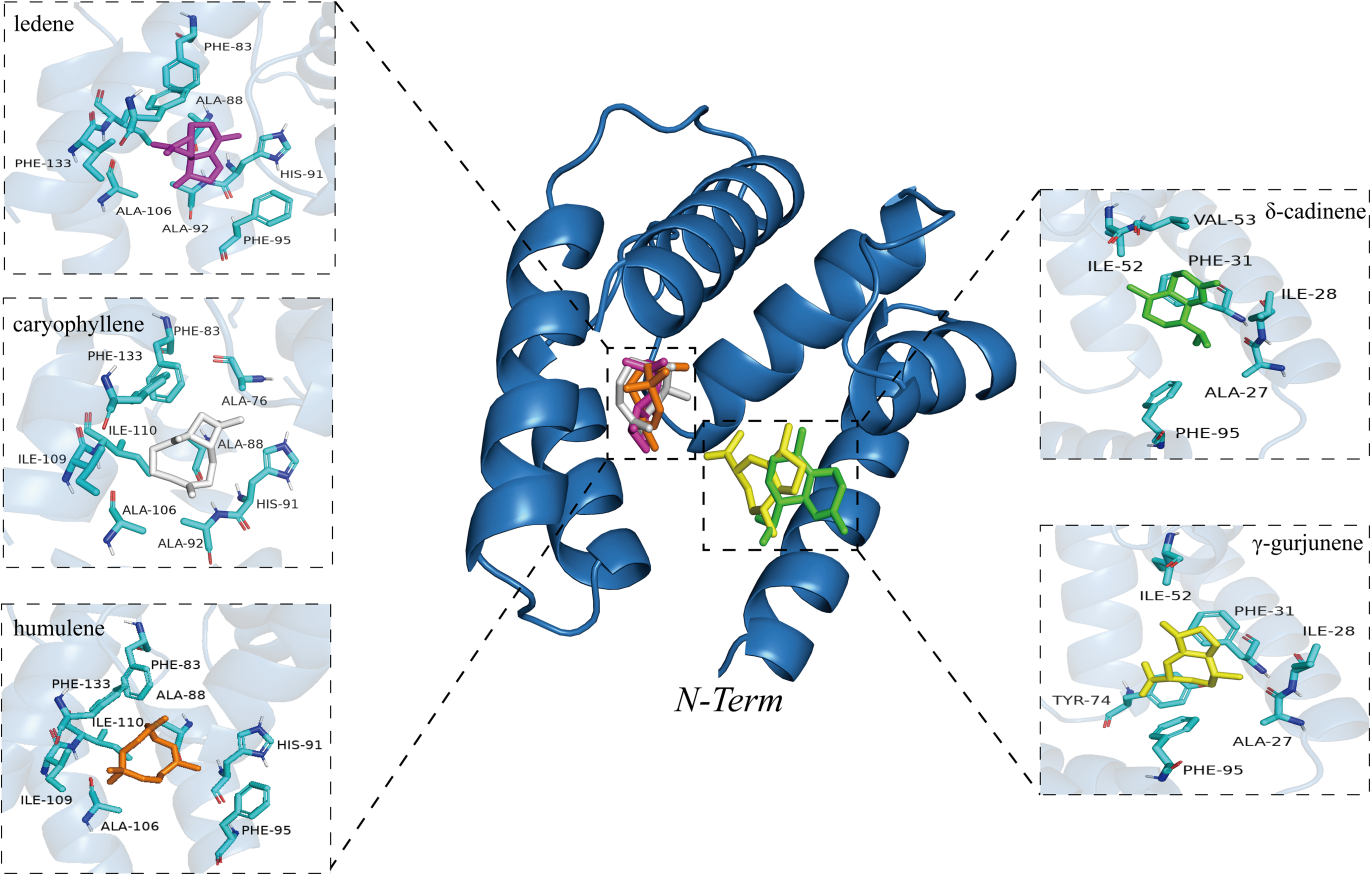
Canonical conformations of BdorOBP84a-1-ligand complexes.

**Figure 5:**
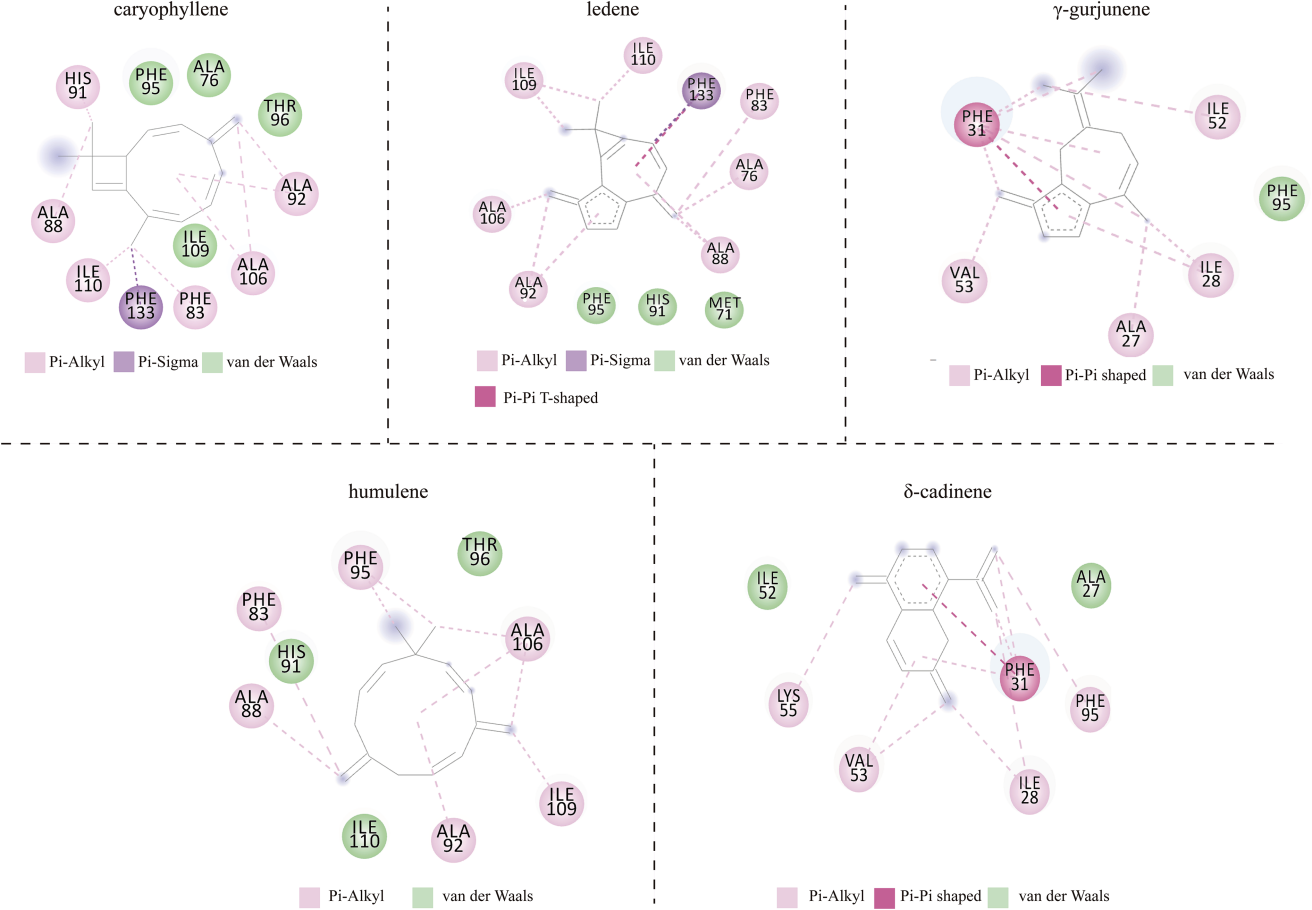
interactions of BdorOBP84a-1-ligand complexes.

The analysis revealed canonical conformations of BdorOBP84a-1-ligand complexes, highlighting key structural determinants of binding (**Figure 4**). Free energy landscape (FEL) analysis was used to explore conformational stability and binding interactions (**Supplementary Figure S1**). The lowest-energy conformations were selected for molecular interaction analysis (**Figure 5**):

BdorOBP84a-1-caryophyllene complex: PHE95, ALA76, TYR96, and ILE109 engaged in van der Waals interactions, while PHE133 formed a Pi-Sigma interaction. HIS91, ALA88, ILE110, PHE83, ALA106, and ALA92 contributed Alkyl and Pi-Alkyl interactions.

BdorOBP84a-1-ledene complex: HIS91, PHE95, and MET71 formed van der Waals contacts, while PHE133 established Pi-Sigma and Pi-Pi T-shaped interactions. Multiple residues (ILE109, ILE110, PHE83, ALA76, ALA106, ALA92, ALA88) participated in Pi-Alkyl interactions.

BdorOBP84a-1-γ-gurjunene complex: PHE95 formed van der Waals contacts, PHE31 engaged in Pi-Pi Stacked interaction, and ILE52, VAL53, ALA27, and ILE28 contributed Alkyl and Pi-Alkyl interactions.

BdorOBP84a-1-humulene complex: THR96, HIS91, and ILE110 formed van der Waals interactions, whereas PHE95, PHE83, ALA88, ALA92, and ILE109 contributed Alkyl and Pi-Alkyl interactions.

BdorOBP84a-1-δ-cadinene complex: ILE52 and ALA27 formed van der Waals contacts, PHE31 engaged in Pi-Pi stacking, and LYS55, VAL53, ILE28, and PHE95 exhibited Alkyl and Pi-Alkyl interactions.

### 3.5 Energy calculation of BdorOBP84a-1-ligand complexes

Binding free energies calculated using the MM/PBSA method (**Table 1**) were -24.38 kcal/mol (δ-cadinene), -23.16 kcal/mol (humulene), -30.62 kcal/mol (ledene), -21.17 kcal/mol (γ-gurjunene), and -31.87 kcal/mol (caryophyllene). The negative values indicate favorable binding, with lower values corresponding to stronger affinities. Notably, in the Ledene complex, the Pi-Pi T-shaped interaction with PHE133 contributed to relatively high electrostatic and gas-phase free energies, though the overall free energy remained comparable to other complexes.

**Table 1:** Free binding energies of BdorOBP84a-1-ligand. (Energy: kcal/mol).

Free binding energy	ligand				
	Caryophyllene	Ledene	γ-Gurjunene	Humulene	δ-Cadinene
Van der Waals Energy	-37.07	-12.19	-36.32	-30.13	-30.89
Electrostatic Energy	0	-111.98	-3.08	1.1	-1.35
Polar Solvation Energy	5.92	96.39	21.53	8.95	10.82
Non-Polar Solvation Energy	-0.72	-2.84	-3.3	-3.08	-2.96
Gas phase free Energy	-37.07	-124.17	-39.4	-29.03	-32.24
Solvation free Energy	5.2	93.55	18.23	5.87	7.86
Binding energy	-31.87	-30.62	-21.17	-23.16	-24.38

**Figure 6:**
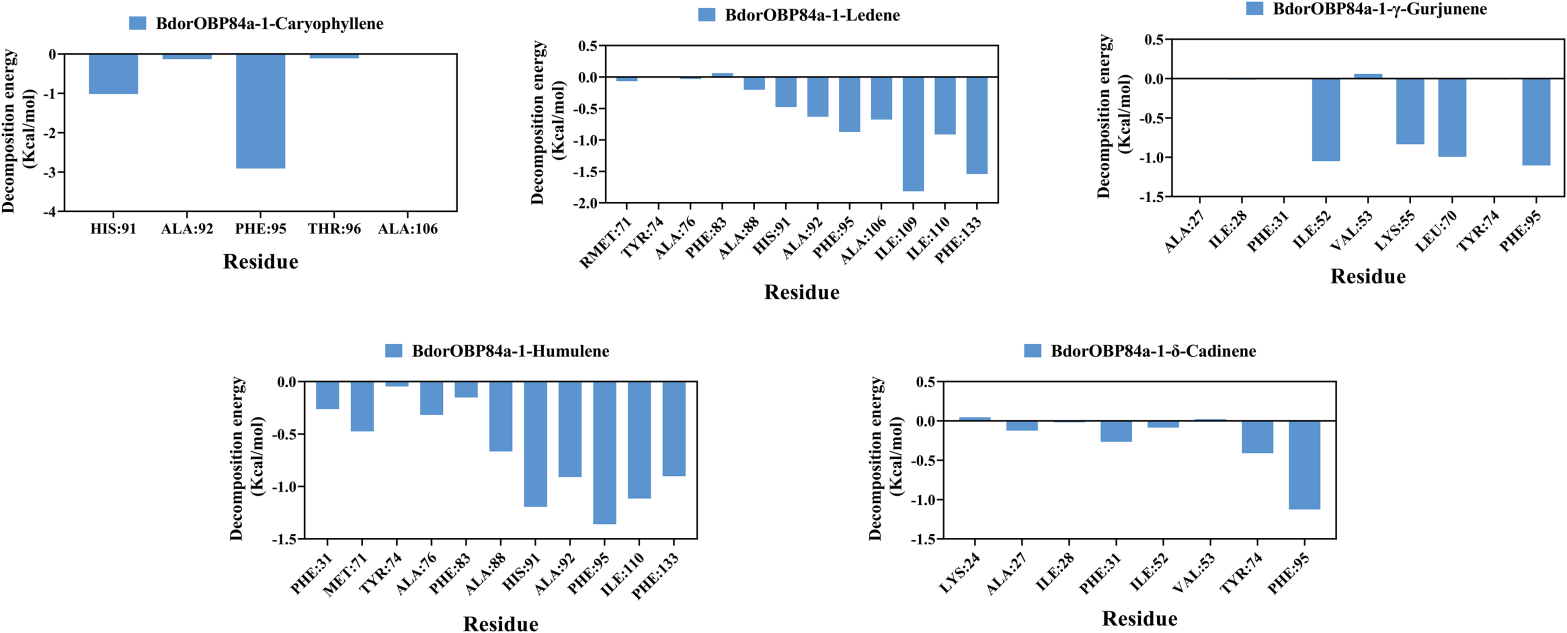
MM-PBSA energy contributions of BdorOBP84a-1-ligand.

Residue energy decomposition analysis identified amino acids with major contributions to ligand binding: Caryophyllene complex (**Figure 6**): PHE95, HIS91, ALA92, THR96. Ledene complex: ILE109, PHE133, ILE110, PHE95, ALA106, ALA92, HIS91, ALA88. γ-Gurjunene complex: PHE95, ILE52, LEU70, LYS55. Humulene complex: PHE95, HIS91, ILE110, PHE133, ALA92, ALA88, MET71, ALA76, PHE31, PHE83. δ-Cadinene complex: PHE95, TYR74, PHE31, ALA27. These residues likely play crucial roles in stabilizing ligand binding and may contribute significantly to the molecular recognition mechanism of BdorOBP84a-1.

### 3.6 Y-tube olfactometry behavioral assay

**Figure 7:**
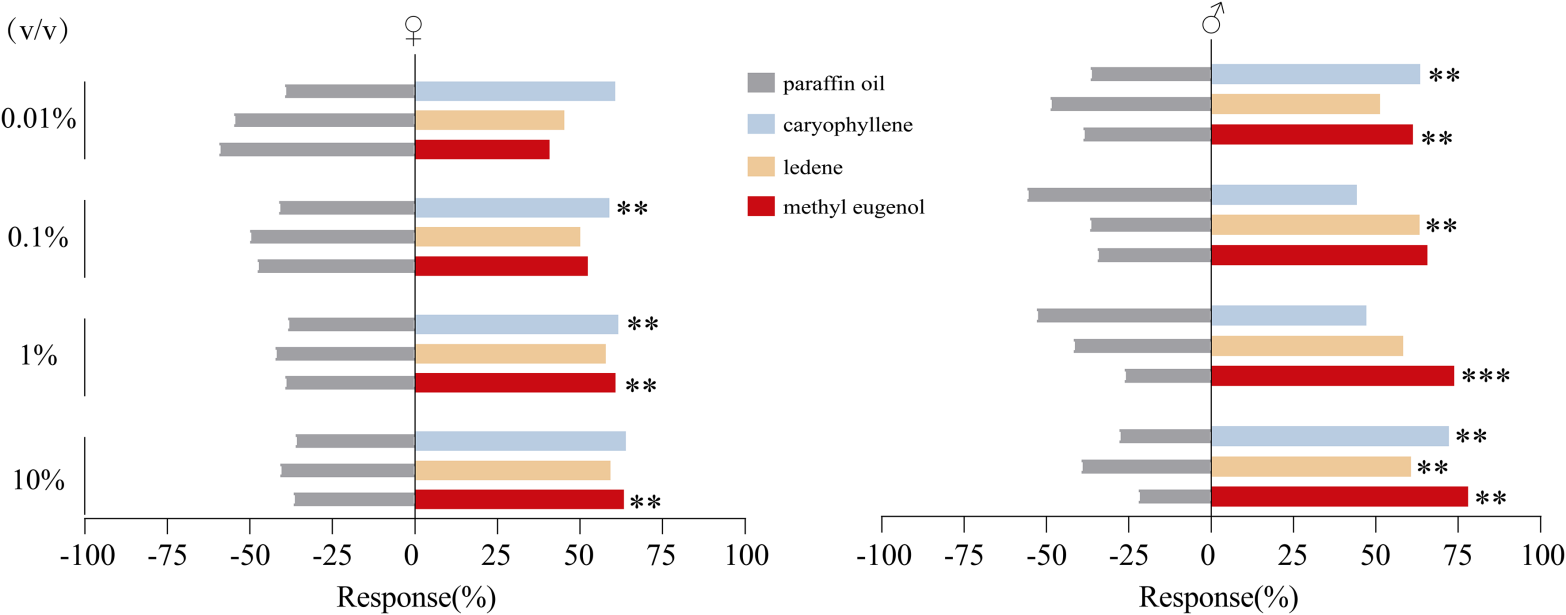
Orientation responses of female and male of *B*. *dorsalis*. Insect choice data were evaluated using binomial tests. Asterisks indicate significance levels: ***p < 0.001; **p < 0.01.

Y-tube olfactometer assays demonstrated that ME, used as a positive control, elicited a clear dose-dependent attraction in male *B. dorsalis*. The proportion of males choosing the treatment arm increased steadily with concentration, reaching 78.01% at 10%. At 1% ME attracted males at highly significant levels (*p* < 0.001), whereas females responded only at 1% and 10%. For caryophyllene, males showed significant attraction at 0.01% and 10%, while females responded more strongly at 0.1% and 1%. In contrast, ledene elicited attraction in males only at 0.1% and 10%, and no significant response was observed in females (**Figure 7**).

## 4 Discussion

*B. dorsalis* is one of the most destructive pests of mango during fruit ripening, and current control strategies still rely heavily on attractants. OBPs are key proteins of the insect olfactory system, they are responsible for the selective recognition of host plant volatiles. Acting as soluble carriers, OBPs solubilize hydrophobic odorants and transport them through the sensillar lymph to receptor neurons. Genomic analyses have identified 49 OBP genes in *B. dorsalis*, six OBP show high antennal expression and are likely involved in olfactory reception (34). Among these, *BdorOBP84a-1* exhibits an antenna specific high expression profile, this suggest a predominant role in olfactory signaling (37),

Consistent with this hypothesis, our study showed that feeding on Guire No.82 mango significantly increased *BdorOBP84a-1* expression. This upregulation corresponded to its strong binding affinity for sesquiterpenes. This observation is supported by previous research showing that several antennal OBPs are specialized for detecting host volatiles. For example, *BdorOBP13* has been cloned and functionally validated for olfactory roles, while receptor-level analyses identified *BdorOR88a* as responsive to methyl eugenol (ME), providing a receptor-end anchor in the attractant pathway (38). At the protein-binding layer, CRISPR/Cas9 knockout of *BdorOBP56f-2* disrupted ME perception, establishing a causal link between an OBP and behavioral sensitivity (39). Beyond olfaction, accumulating evidence indicates that OBPs participate in a wide range of physiological processes. For example, *BdorOBP28a-2* expressed in the legs enhances malathion tolerance (40). These findings suggest that OBPs expressed outside the antennae may buffer exogenous hydrophobic molecules and contribute to detoxification, complementing their well-recognized ligand-binding versatility. Other OBPs have been implicated in nutrient and amino-acid detection (41), humidity sensing (42), and even innate immunity through the Toll pathway (43). This evidence supports the view that OBPs function as multifunctional proteins rather than being restricted to odorant transport.

Guire No.82, also known as Guiqi mango, possesses a distinct aromatic profile (44). GC-MS analysis of ten Chinese mango cultivars revealed that Guire No.82 contained particularly high levels of caryophyllene (81.07 μg/kg), far exceeding those of other cultivars. Notably, ledene was exclusively detected in in this cultivar, whereas caryophyllene is also a volatile component of other *B. dorsalis* host plants, including wax apple, murraya, guava, and bayberry (45). We observed weak binding affinity of humulene to BdorOBP84a-1, and this finding is consistent with earlier reports showing that both caryophyllene and humulene can significantly attract *B. dorsalis* (46).

To explore these interactions further, we employed computational and structural approaches. In protein-ligand interaction research, fluorescence binding assays and molecular docking are widely used and often yield results consistent with experimental data (47). Nevertheless, docking results are inherently constrained by receptor model quality. Lower-resolution structures can reduce confidence in predicted interactions. Here, the use of AlphaFold3 significantly improved structural accuracy, providing physically plausible templates for docking and subsequent MD simulations (48). AF3-based models of BdorOBP84a-1 exhibited the canonical six-helix fold stabilized by three disulfide bonds, which follows the classic OBP structure. This greatly enhances the reliability of docking and free-energy calculations, thereby overcoming one of the main limitations of conventional in silico approaches (16).

Docking analyses identified caryophyllene and ledene as the strongest ligands of BdorOBP84a-1. To further validate these predictions, we conducted 100-ns molecular dynamics (MD) simulations. Evaluation of RMSD and RMSF values showed stable binding complexes with compact conformations (49). Similar results have been reported in other insects, such as GOBP1 and GOBP2 in *Glyphodes pyloalis*, which demonstrated that ligand binding led to stable β-ionone and hexadecanal with sustained RMSD plateaus and reduced local flexibility, suggesting structurally stable interactions (50). The resulting RMSD, RMSF, Rg, SASA and free energy landscape (FEL) analyses confirmed stable complexes and well-defined energy minima. Except for the γ-gurjunene complex, all tested systems stabilized after 65–70 ns, with most residues showing RMSF values <2 Å. Sesquiterpenes such as ledene, humulene, δ-cadinene, and caryophyllene consistently formed tightly packed, low-flexibility complexes. The agreement between docking rankings and MD free-energy profiles underscores the value of combining both methods as best practice for OBP-ligand studies (11).

Binding free energies were estimated using MM/PBSA calculations (51). Caryophyllene (-31.87 kcal mol^−1^) and ledene (-30.62 kcal mol^−1^) ranked highest, followed by δ-cadinene, humulene, and γ-gurjunene. Residue-based energy decomposition identified PHE95, HIS91 and PHE133 as major contributors to binding. A similar pattern was reported in Lepidoptera. In these insects, hydrophobic residues, especially phenylalanine, form binding pockets that stabilize ligands (15). They are frequently stabilized terpenoid ligands through van der Waals and Pi-sigma interactions. For caryophyllene and ledene, these interactions resulted in particularly compact complexes, while γ-gurjunene relied mainly on weaker van der Waals forces, producing less stable conformations (52). Such aromatic and aliphatic side chains have been widely recognized as anchors for terpenoid recognition across insect taxa (37). In *Adoxophyes orana*, Z9-14:Ac interacts with the phenyl rings of Phe36 and Phe118 in AoraGOBP1 and with Phe13 in AoraGOBP2 through Pi-Sigma interactions (53). Functionally, mutating these residues would be expected to weaken sesquiterpene binding, thereby potentially diminishing antennal sensitivity to host-derived volatiles. This provides a mechanistic framework linking structural determinants to ecological function.

Beyond structural validation, behavioral assays confirmed the biological relevance of our findings. In Y-tube bioassays, ME strongly attracted males at higher concentrations, while females showed weaker and concentration-dependent, consistent with previous reports (38, 54). Caryophyllene elicited sex and dose-specific responses. Males were attracted at very 0.01% and 10% concentrations, while females responded mainly at intermediate doses (46, 55). Similar patterns have been observed in guava and citrus, where low concentrations of caryophyllene were sufficient to attract females (55, 56). These trends are consistent with the well-established role of fruit volatiles in generating concentration-dependent behavioral activity. Ledene showed limited effects, attracting only males at 0.1% and 10%, suggesting restricted sex-specific relevance. Over all, our results highlight the importance of concentration in shaping olfactory responses. While ME remains the most effective lure, caryophyllene also trigger significant behavioral activity. These compounds may serve as complementary attractants if optimized in blends and concentrations for pest management.

This study highlights the strong binding of caryophyllene and ledene to BdorOBP84a-1, but the conclusions are mainly supported by expression analysis and computational modeling. To further substantiate the binding activity of BdorOBP84a-1, future work should include additional *in vitro* binding assays and behavioral experiments following the silencing of this protein, which would help verify its affinity for methyl eugenol and its involvement in detecting attractive VOCs. In addition, field trials will be essential to validate the predicted binding activities and evaluate the practical performance of candidate lures. Together, these efforts will help translate molecular insights into effective, pest-targeted monitoring and control strategies for *B. dorsalis*.

## 5 Conclusions

This study shows that BdorOBP84a-1 is a key protein for sesquiterpene recognition in *B. dorsalis*. Structural modeling confirmed binding of caryophyllene and ledene. MD simulations showed the binding was strong and stable, supported by hydrophobic and aromatic residues. Behavioral assays revealed clear dose-dependent attraction. Caryophyllene attracted both males and females, with males responding at very low and high doses, and females responding more strongly at intermediate doses. Ledene attracted males at 0.1% and 10% but did not affect females. These results suggest that caryophyllene and ledene are potential attractants and may complement methyl eugenol in pest management.

## Data Availability

The original contributions presented in the study are included in the article/**Supplementary Material**. Further inquiries can be directed to the corresponding author.
