# Supplementary figures and images for: Odorant-binding protein 84a-1 mediates detection of Guire No.82 mango volatiles in Bactrocera dorsalis: from structural analysis to behavioral validation
Source: Front Insect Sci. 2026 Jan 6;5:1712208. doi: 10.3389/finsc.2025.1712208 (PMC12815875; doi:10.3389/finsc.2025.1712208)

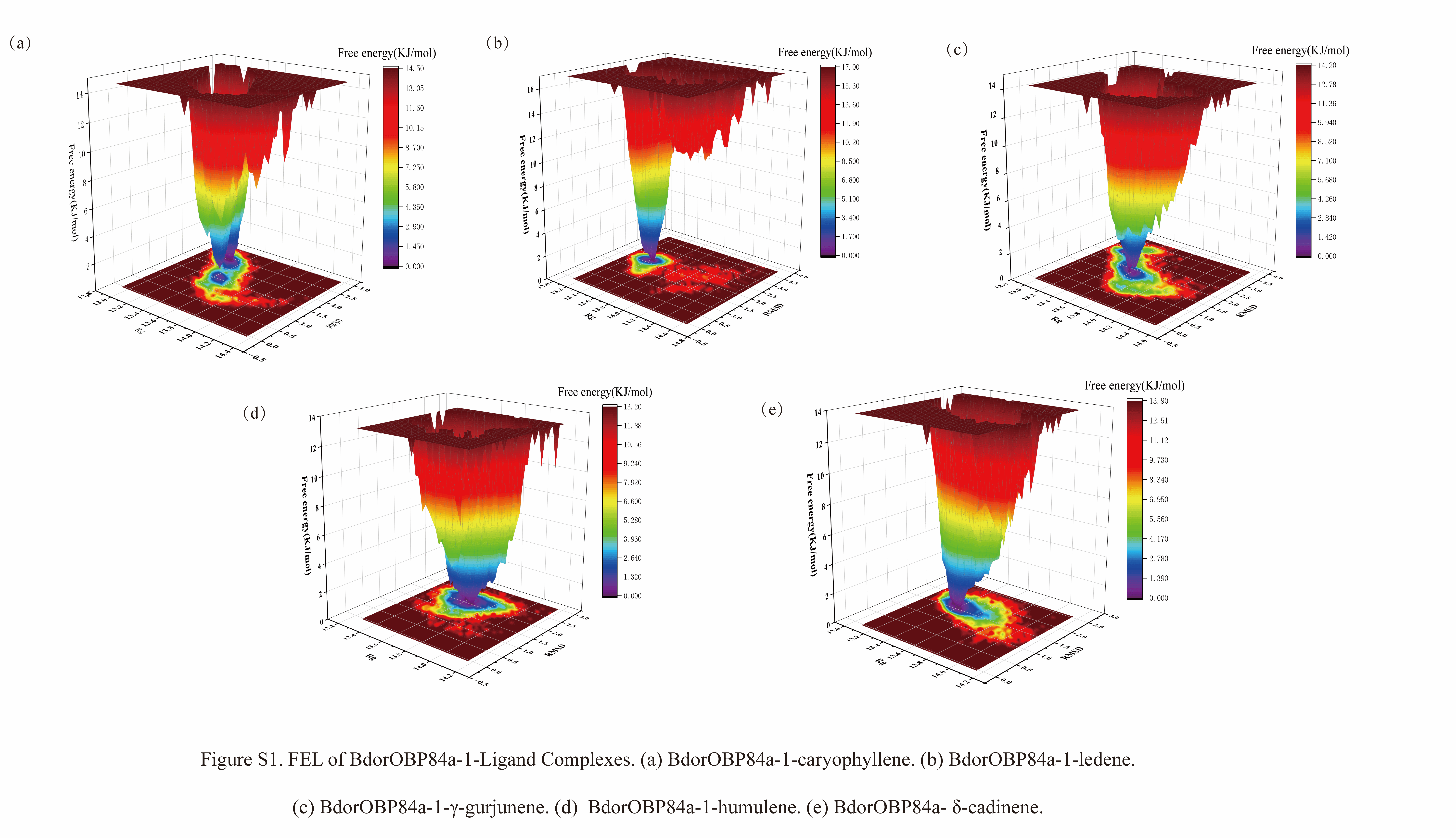

Supplement: Supplementary file 3 [file Image1.tif]
